# Impact of height difference between coronary ostium and location of intracoronary pressure sensor on fractional flow reserve measurements

**DOI:** 10.1371/journal.pone.0289646

**Published:** 2023-08-24

**Authors:** Moon-Seung Soh, Hangyul Kim, Min Gyu Kang, Hyo Jin Lee, Seung Do Lee, Seok-Jae Hwang, Jin-Yong Hwang, Kyehwan Kim, Jeong-Rang Park, Hye-Ree Kim, Seung-Jea Tahk, Myeong-Ho Yoon, Hong-Seok Lim, Jin-Sin Koh

**Affiliations:** 1 Department of Cardiology, Ajou University School of Medicine, Suwon, Republic of Korea; 2 Division of Cardiology, Department of Internal Medicine, Gyeongsang National University School of Medicine, Gyeongsang National University Hospital, Jinju, Republic of Korea; BSMMU: Bangabandhu Sheikh Mujib Medical University, BANGLADESH

## Abstract

**Background:**

During fractional flow reserve (FFR) measurements, distal coronary pressure (Pd) can be influenced by hydrostatic pressure changes resulting from the height difference (HD) between the coronary ostium and the location of the distal pressure sensor.

**Aims:**

We investigated the effect of aortocoronary HD on the FFR measurements in each coronary artery.

**Methods:**

In this retrospective cohort study, we analyzed 257 patients who underwent FFR measurements and coronary computed tomography (CCTA) within a year. Using CCTA, we measured HD as the vertical distance between the coronary ostium and a matched point of the distal coronary pressure sensor identified on coronary angiography.

**Results:**

The location of the Pd sensor was higher than the coronary ostium in the left anterior descending artery (LAD) (-4.64 ± 1.15 cm) and lower than the coronary ostium in the left circumflex artery (LCX) (2.54 ± 1.05 cm) and right coronary artery (RCA) (2.03 ± 1.28 cm). The corrected FFR values by HD were higher in the LAD (0.78 ± 0.09 to 0.82 ± 0.09, P<0.01) and lower in the LCX and RCA than the original FFR values (0.87 ± 0.07 to 0.85 ± 0.08, P<0.01; 0.87 ± 0.10 to 0.86 ± 0.10, P<0.01, respectively). Using an FFR cut-off value of 0.8, the concordance rates between the FFR and corrected FFR values were 77.8%, 95.2%, and 100% in the LAD, LCX, and RCA, respectively.

**Conclusion:**

HD between the coronary ostium and the distal coronary pressure sensor may affect FFR measurements and FFR-guided treatment decisions for coronary artery disease.

## Introduction

Fractional flow reserve (FFR) is the most verified diagnostic tool for evaluating lesion-specific myocardial ischemia caused by coronary stenosis identified by coronary angiography (CAG) [1–4]. The operator determines whether to perform coronary revascularization based on the result of FFR. The FFR is calculated as the ratio of distal coronary pressure (Pd) to aortic pressure (Pa) during maximum hyperemia. At the beginning of the FFR measurement procedure, it is mandatory to equalize the pressure between the Pd sensor of the guidewire and Pa to obtain accurate FFR values [5, 6].

However, after pressure equalization at the ostium, Pd can be influenced by hydrostatic pressure variations related to the height difference (HD) between the coronary ostium and the distal coronary pressure sensor. The vertical location of the pressure sensor of the wire can change with wire advancement according to the coronary anatomy in the supine position during the procedure [7, 8]. Therefore, it has been suggested that HD may affect FFR measurement. Previous animal and human studies have reported interference of FFR by hydrostatic pressure with HD, and accurate FFR values were obtained with HD correction [9, 10]. In this study, we aimed to determine the effect of HD on FFR measurements and its clinical significance in actual clinical practice.

## Methods

### Patient population

**Fig 1:**
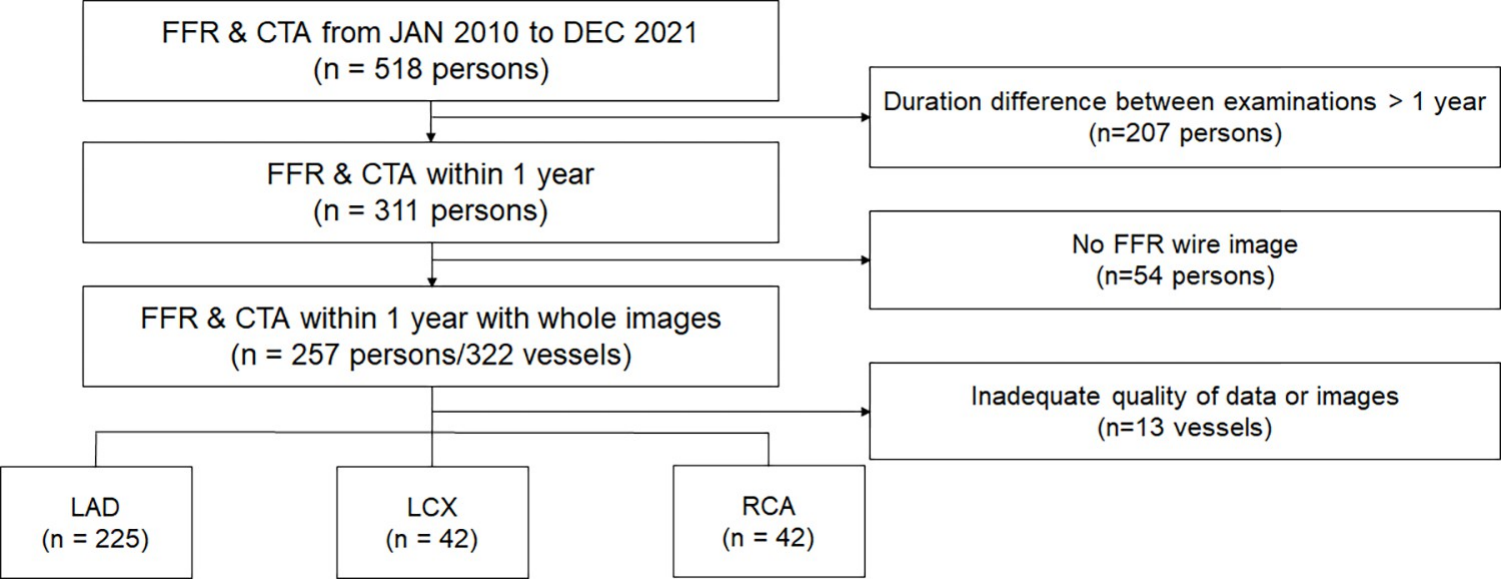
Flow chart. Patients who underwent FFR measurements and coronary computed tomography angiography within a year between January 2010 and December 2021 were enrolled. Patients with an inter-examination period >1 year (n = 207), those in whom the position of the pressure wire could not be identified from the image (n = 54), and those with unavailable physiological values (n = 13) were excluded. A total of 257 patients and 309 vessels were analyzed. CTA, computed tomography angiography; FFR, fractional flow reserve; LAD, left anterior descending artery; LCX, left circumflex artery; RCA, right coronary artery.

From January 2010 to December 2021, patients who underwent FFR measurement and coronary computed tomography angiography (CCTA) within a year in two independent cardiovascular centers in Korea were pooled retrospectively. Patients with left main disease, in-stent restenosis, coronary bypass surgery, FFR in the infarct-related artery, inadequate data quality, or CCTA images were excluded (Fig 1). In this study, we have taken care to appropriately handle and protect any identifiable personal information of the participants. All such information was anonymized during the research process, ensuring both the protection of participant privacy and the ethical conduct of our study. Notably, the authors did not have access to any information that could identify individual participants during or after data collection. The study protocol was approved by the Institutional Review Board of each hospital (AJOUIRB-MDB-2022-250 and GNUH 2021-12-007). The Ethics Committee waived the requirement for written informed consent, because of the study’s retrospective nature.

### Quantitative coronary angiographic analysis and fractional flow reserve measurement

Quantitative coronary angiography (QCA) was performed at each hospital with blinding. Reference diameter, minimum lumen diameter, lesion length, and percent diameter stenosis were measured using a coronary catheter for calibration and an edge detection system (CAAS II, Pie Medical, Maastricht, Netherlands and Medis Suite 4.0, Medis MEDICAL IMAGING, Netherlands). The reference diameter was determined using an interpolated reference method. FFR was measured using a 0.014-inch pressure guidewire (Abbott Vascular Inc. Santa Clara, CA, USA) according to the standard method, as previously described [6]. After equalization of pressures, with the Pd sensor positioned at the guiding catheter tip, the Pd sensor was advanced distally to a target lesion. After recording the baseline distal intracoronary pressure, maximum hyperemia was induced using one of the following agents: 1) intravenous adenosine (140 g/(kg·min)), 2) intracoronary adenosine bolus injection (80 μg for the left coronary artery or 40 μg for the right coronary artery), or 3) intracoronary nicorandil bolus injection (2 mg). The procedure manual of each hospital recommended the Pd sensor to be placed at least 3 cm distal to the stenotic lesion. FFR was calculated as the ratio of Pd to Pa during maximum hyperemia.

### Coronary computed tomography angiography and height difference

**Fig 2:**
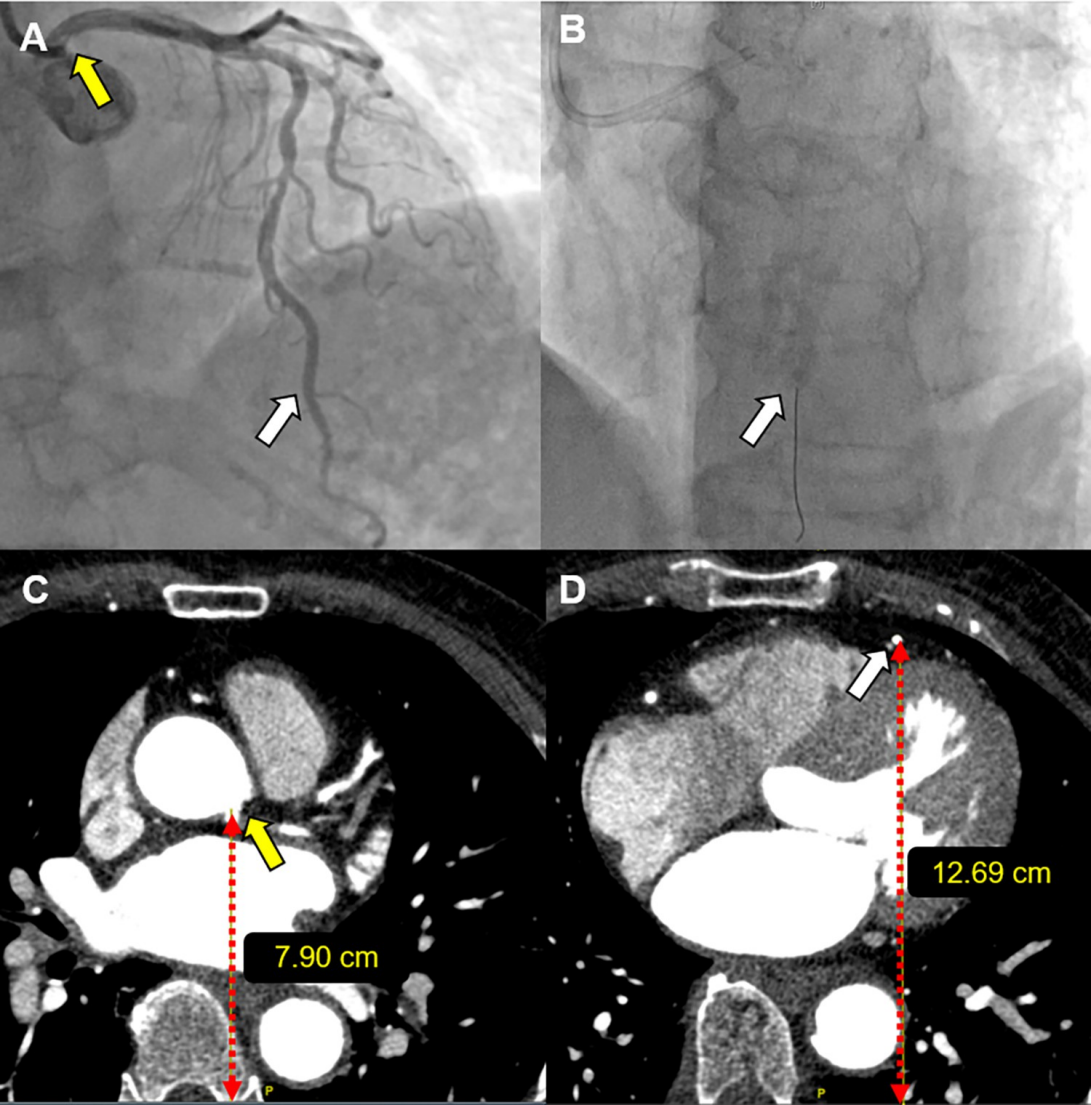
Measurement of aortocoronary height difference. Representative case of an 80-year-old woman with typical angina. Coronary angiography showed 50% stenosis in the mid left anterior descending artery on the anterior-posterior-cranial view (A). The pressure wire was placed as distal as possible. A small diagonal branch next to the pressure sensor was the landmark for heightening the wire (white arrow) (B). The height was measured from the base of the computed tomography (CT) image to the left main ostium (yellow arrow) (C). Then, the landmark (small diagonal branch) was traced, and the height was measured from the base of the CT image (D). The difference between the two measurements can be used to estimate the height from the ostium to the pressure sensor. CT, computed tomography.

CCTA was performed according to common standards, using computed tomography (CT) scanners with at least 64 slices (SOMATOM Force 192 channel, Siemens Healthineers, Forchheim, Germany; Ingenuity Core 128, Philips, Amsterdam, Netherlands; SOMATOM Definition Flash, Siemens Healthineers, Forchheim, Germany). All CT images were collected at the central workstation of each hospital (Syngo via Client, TERRARECON AQNET, US) and analyzed using an application (General Electric PACS system, US) by researchers blinded to the study. The CAG and CCTA data of each patient were compared to analyze the HD between the coronary ostium and pressure sensor of the intracoronary pressure wire. Using frame-by-frame CT image tracing, the points of the coronary ostium and the location of the distal pressure sensor were identified by landmarks such as side branches and vessel curves matched with the CAG. The height from the bottom to the coronary ostium and the location of the distal coronary pressure sensor were measured vertically. The HD was calculated as the difference between the heights (Fig 2). To minimize potential bias, data from each institution was rigorously cross-checked, and two cardiologists measured height difference.

### Correction of fractional flow reserve by hydrostatic pressure

Previous studies demonstrated that the absolute pressure difference correlated strongly with the HD, and the slope was 0.77 mmHg/cm by HD [9]. The corrected Pd was calculated as Pd + 0.77 × HD (cm), and the corrected FFR was defined as the ratio of corrected Pd to Pa during maximum hyperemia. This correction method was adapted from a previous study using the mercury (13.55 g/cm^3^): blood (1.05 g/cm^3^) specific gravity ratio [7].

### Statistical analysis

Continuous variables are expressed as means and standard deviations. Categorical variables are expressed as numbers. The comparison of resting Pd/Pa and FFR values was performed using the Student’s t-test. The original and corrected FFR values were presented as scatter plots, and the differences between the two FFR values were evaluated using a paired t-test. Missing data were handled using listwise or pairwise deletion, where incomplete cases were removed from the analysis. All statistical analyses were performed using Statistical Package for Social Sciences ver. 26 for Windows (SPSS Inc., Chicago, IL, USA) and Prism GraphPad 9^th^ (Dotmatics, San Diego, CA, USA). Statistical significance was set at p <0.05.

## Results

### Baseline characteristics

A total of 257 patients with 309 vessels were enrolled in this study (Fig 1). Table 1 shows the baseline clinical characteristics of the patients. The mean age of the patients was 66 ± 9 years, and 73.9% were male. Diabetes and hypertension were observed in 83 (32.3%) and 158 patients (61.5%), respectively. Among these, 116 patients (45.1%) had stable angina, and 63 (24.5%) were diagnosed with acute coronary syndrome.

**Table 1:** Baseline characteristics.

Clinical characteristics	Total (n = 257)
Age, years	65.9 ± 9.3
Male, n (%)	190 (73.9%)
Systolic blood pressure (mmHg)	135.1 ± 20.1
Currently smoking, n (%)	27 (10.5%)
Old myocardial infarction, n (%)	20 (7.8%)
Previous PCI, n (%)	39 (15.2%)
Previous coronary artery bypass graft, n (%)	1 (0.4%)
Hypertension, n (%)	158 (61.5%)
Diabetes, n (%)	83 (32.3%)
Dyslipidemia, n (%)	114 (46.2%)
Chronic kidney disease, n (%)	16 (6.2%)
Stroke, n (%)	22 (8.6%)
Laboratory data	
Total cholesterol (mg/dL)	151.5 ± 41.7
HDL (mg/dL)	49.2 ± 13.1
LDL (mg/dL)	81.1 ± 36.6
LVEF (%)	64.5 ± 9.8
Indication for CAG, n (%)	
Stable angina	116 (45.1%)
Acute coronary syndrome	63 (24.5%)
Abnormal findings in non-invasive studies	63 (24.5%)
Heart failure	5 (1.9%)
Follow-up coronary angiography	4 (1.6%)
Other	6 (2.3%)
Medication, n (%)	
Antiplatelets	130 (52.6%)
Statins	155 (62.8%)
RAS inhibitors	121 (49.2%)
Calcium channel blockers	78 (31.6%)
Beta blockers	42 (17.0%)
Vasodilators	41 (16.6%)
Oral hypoglycemic agents	76 (30.8%)

Data are presented as mean ± standard deviation or n (%). CAG, coronary angiography; PCI, percutaneous coronary intervention; LDL, low-density lipoprotein; HDL, high-density lipoprotein; LVEF, left ventricular ejection fraction; RAS, renin-angiotensin system

### Angiographic characteristics, height difference, and fractional flow reserve

**Fig 3:**
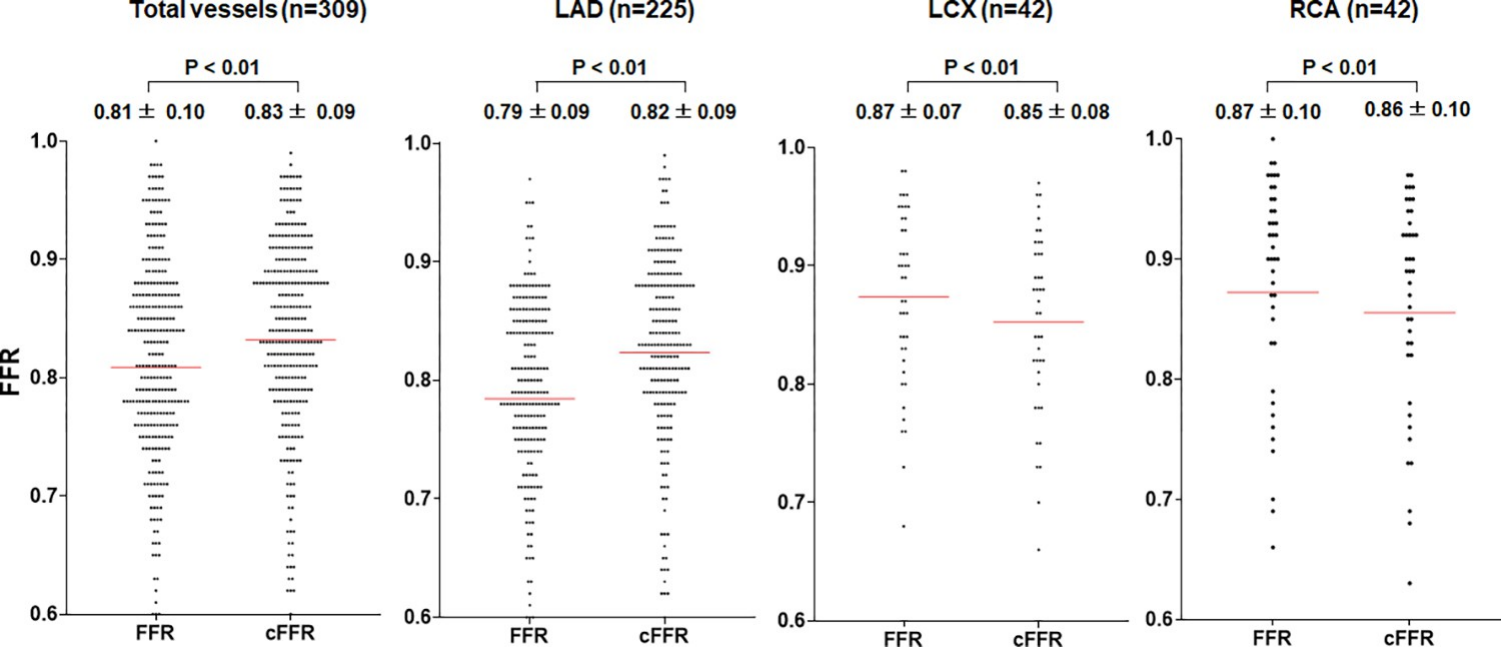
FFR and corrected FFR values. After correction according to the height difference, the overall FFR value increased. In the LAD, the corrected FFR value increased, while in the LCX and RCA, it decreased. FFR, fractional flow reserve; LAD, left anterior descending artery; LCX, left circumflex artery; RCA, right coronary artery.

**Fig 4:**
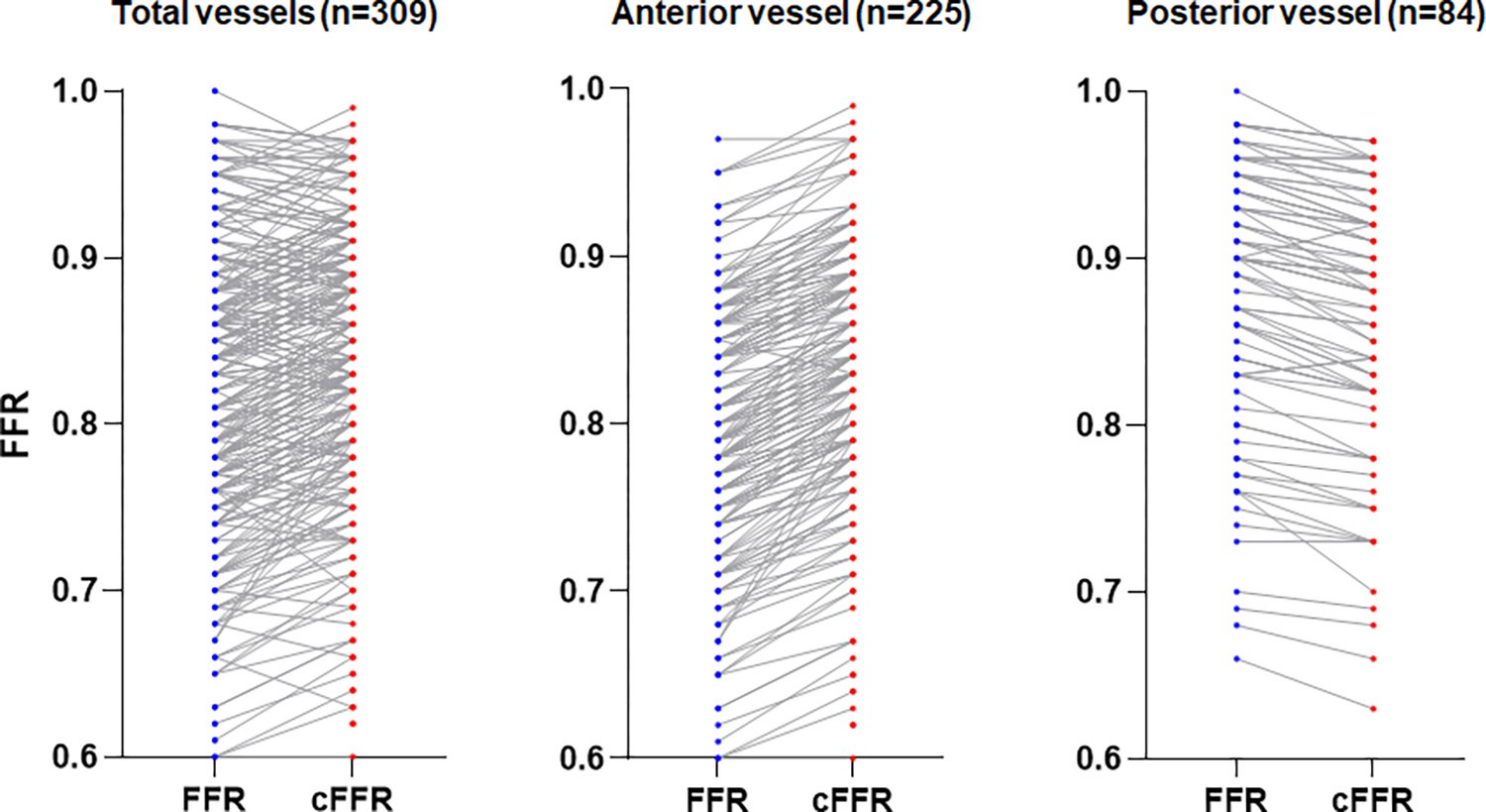
Comparison of FFR and corrected FFR values. Overall, FFR values in the anterior coronary vessels tended to increase, whereas those in the posterior coronary vessels tended to decrease after FFR correction by height difference. FFR, fractional flow reserve; cFFR, corrected fractional flow reserve; FFR, fractional flow reserve; HD, height difference; LAD, left anterior descending artery; LCX, left circumflex artery; RCA, right coronary artery.

Table 2 presents the angiographic findings, HD, and physiological measurements. The left anterior descending artery (LAD) (72.8%) was the most frequently examined coronary artery. The mean diameter stenosis and lesion length of all vessels were 58.9% and 22.8 mm, respectively. The mean FFR value was 0.81 ± 0.10. The FFR values of the LAD, left circumflex artery (LCX), and right coronary artery (RCA) were 0.78 ± 0.09, 0.87 ± 0.07, and 0.87 ± 0.10, respectively. The mean corrected FFR value was 0.83 ± 0.09. The corrected FFR values of the LAD, LCX, and RCA were 0.82 ± 0.09, 0.85 ± 0.08, and 0.86 ± 0.10, respectively. Among all three vessels, the LAD had the largest HD (-4.64 ± 1.15 cm). The FFR value of the LAD increased after HD adjustment (0.78 ± 0.09 to 0.82 ± 0.09, P-value<0.01), while the FFR values of the LCX (0.87 ± 0.07 to 0.85 ± 0.08, P-value<0.01) and RCA (0.87 ± 0.10 to 0.86 ± 0.10, P-value<0.01) decreased (see Figs 3 and 4).

**Table 2:** Quantitative coronary angiography, height difference, and physiologic measurements.

		LAD (n = 225)	LCX (n = 42)	RCA (n = 42)	
Quantitative coronary angiography					P-value
	Diameter stenosis (%)	60.3 ± 11.5	55.4 ± 10.4	54.3 ± 10.0	0.001
	Reference vessel diameter (mm)	3.11 ± 2.02	2.83 ± 0.54	3.11 ± 0.83	0.479
	Minimum lumen diameter (mm)	1.17 ± 0.41	1.26 ± 0.41	1.40 ± 0.49	0.019
	Lesion length (mm)	23.49 ± 12.38	21.62 ± 12.81	20.21 ± 13.07	0.178
Aortocoronary height difference (cm)		-4.64 ± 1.15	2.54 ± 1.05	2.03 ± 1.28	<0.001
Physiologic measurements					
	Pa (mmHg)	99.2 ± 14.8	100.2 ± 13.3	101.1 ± 12.4	0.503
	Pd (mmHg)	90.2 ± 15.1	96.7 ± 16.1	98.3 ± 13.2	<0.001
	Pd/Pa	0.91 ± 0.06	0.96 ± 0.06	0.97 ± 0.06	<0.001
	Hyperemic Pa (mmHg)	93.8 ± 15.6	90.8 ± 14.3	92.4 ± 13.4	0.551
	Hyperemic Pd (mmHg)	73.8 ± 14.7	79.6 ± 14.9	80.5 ± 14.5	0.002
	FFR	0.78 ± 0.09	0.87 ± 0.07	0.87 ± 0.10	<0.001
	Corrected Pd (mmHg)	93.7 ± 15.0	94.8 ± 16.3	96.7 ± 13.3	0.389
	Corrected Pd/Pa	0.94 ± 0.06	0.94 ± 0.06	0.96 ± 0.06	0.153
	Corrected hyperemic Pd (mmHg)	77.2 ± 14.5	77.7 ± 15.0	78.9 ± 14.5	0.614
	Corrected FFR	0.82 ± 0.09	0.85 ± 0.08	0.86 ± 0.10	0.005

Data are presented as mean ± standard deviation. FFR, fractional flow reserve; LAD, left anterior descending artery; LCX, left circumflex artery; RCA, right coronary artery; Pa, proximal coronary ostium pressure; Pd, distal coronary pressure

### Fractional flow reserve and corrected fractional flow reserve across the cut-off value

Comparison between FFR and corrected FFR values according to the current treatment decision cut-off of 0.8 demonstrated that the concordance rates of the two values were 77.8%, 95.2%, and 100% for the LAD, LCX, and RCA, respectively. In the LAD, 50 out of 127 cases (39.4%) with FFR values ≤0.8 were found to have discordant corrected FFR values >0.8, but no cases were found with FFR values >0.8 and corrected FFR values ≤0.8. In the LCX, there were no discordant cases with FFR ≤0.8 and corrected FFR >0.8, but two cases (5.9%) with FFR >0.8 and corrected FFR ≤0.8. There were no discordant cases in the RCA (Table 3).

**Table 3:** Concordance between FFR and corrected FFR values.

	LAD (n = 225)		LCX (n = 42)		RCA (n = 42)	
	FFR ≤0.8	FFR >0.8	FFR ≤0.8	FFR >0.8	FFR ≤0.8	FFR >0.8
cFFR ≤0.8	77	0	8	2	10	0
cFFR >0.8	50	98	0	32	0	32
	Concordance = 77.8%		Concordance = 95.2%		Concordance = 100%	
	Discordance = 22.2%		Discordance = 4.8%		Discordance = 0%	

cFFR, corrected fractional flow reserve; FFR, fractional flow reserve; LAD, left anterior descending artery; LCX, left circumflex artery; RCA, right coronary artery.

### Pd/Pa and corrected Pd/Pa

The corrected Pd/Pa value was calculated using HD. The mean Pd/Pa value was 0.91 ± 0.06, 0.96 ± 0.06, and 0.97 ± 0.06 in the LAD, LCX, and RCA, respectively. After HD adjustment, the Pd/Pa value of the LAD increased to 0.94 ± 0.06 (P-value <0.001), while the Pd/Pa value of the LCX and RCA decreased to 0.94 ± 0.06 and 0.96 ± 0.06, respectively (both with P-value <0.001). When the Pd/Pa and corrected Pd/Pa values were compared based on a cut-off value of 0.93, the concordance rates between the two values were 71.3%, 97.1%, and 100% in the LAD, LCX, and RCA, respectively.

## Discussion

This study yielded several relevant findings. Firstly, the intracoronary pressure measurements were influenced by HD between the coronary ostium and the Pd sensor varied depending on the coronary anatomy. Secondly, in most cases of the LAD, the Pd sensor was located higher than the ostium, resulting in a negative HD value. Conversely, in the LCX and RCA, the Pd sensor was located lower than the ostium, resulting in a positive HD value in most cases. Lastly, the corrected FFR value calculated using the corrected Pd according to the HD showed a significant difference from the original FFR value. Based on the FFR cut-off value (0.8 for treatment decisions), the discordance rate between the two values was 16.8%.

The HD may vary depending on the path of the coronary vessels. The LAD has an upward course, whereas the LCX has a downward course. The RCA initially takes an upward course, runs horizontally, and then takes a downward course [8, 11]. Due to this structural difference in the supine position, the distal LAD has a negative HD value, while the LCX and RCA have positive HD values [9]. The hydrostatic pressure by HD of each coronary vessel is different, and the posterior coronary vessel may have a relatively higher FFR value than the anterior coronary vessel [7, 12]. Even in disease-free coronary vessels, the hydrostatic effect causes artifacts in FFR values higher than 1.00, most commonly in posterior vessels of the LCX and RCA in clinical practice [13].

Our study demonstrated that hydrostatic pressure related to HD can affect FFR measurements. There was a significant difference between the FFR and corrected FFR values, with FFR values increasing significantly in the LAD but decreasing in the LCX and RCA when HD was adjusted. The prominent HD and lower rate of concordance between FFR and corrected FFR values in the LAD than in the LCX or RCA suggest that the LAD is the vessel most affected by HD during FFR measurements. Interestingly, in cases where the original FFR value exceeded 0.8, the corrected FFR value never changed to less than 0.8. Therefore, FFR-guided defer is indeed safe in the LAD. In contrast, when the FFR value was less than 0.8, the corrected FFR value changed to equal or more than 0.8 in approximately 40% of the LAD cases.

Previous studies have shown that FFR measurement is confounded between the anterior and posterior vessels due to structural differences in each coronary artery [14, 15]. Furthermore, a discrepancy between the severity of stenosis on CAG and functional evaluations, such as FFR, was more common in the LAD [16–18]. Many studies comparing functional and anatomical assessments of coronary stenosis using FFR and coronary angiography or intravascular imaging showed significant anatomical–functional discordance. Although previous explanations for functional information using anatomical features have not yielded satisfactory results [19–21], the LAD has been consistently identified as an independent predictor of functional significance based on FFR throughout the studies [22], which could be due to its larger myocardial mass, requiring the highest amount of coronary blood flow [23, 24]. However, our study suggests that HD can be added as a plausible explanation for relatively lower FFR values in the LAD than in the LCX and RCA [25, 26].

Several studies have demonstrated that non-hyperemic pressure ratios (NHPRs), such as the instantaneous wave-free ratio, which are derived from pressure measurements taken at rest without adenosine administration, have diagnostic accuracy comparable to that of FFR in determining ischemia [25, 26]. In this study, we observed similar tendencies between Pd/Pa and corrected Pd/Pa by HD as observed in the FFR and corrected FFR values for each coronary artery. These findings suggest that HD can affect both FFR and NHPRs. Further studies are required to investigate the impact of HD on the NHPRs.

Multiple prior studies have substantiated that FFR-guided percutaneous coronary intervention (PCI) is more cost-effective than angio-guided PCI. This is primarily attributed to the reduction in unnecessary PCI procedures, which is a key factor in enhancing cost-effectiveness [27, 28]. In this study, we confirmed that height differences could influence FFR measurements in real-world practice. We can selectively perform PCI to exclusively "truly significant" coronary stenosis by applying HD-corrected FFR in clinical practice. In this regard, it is believed that HD-corrected FFR can further enhance cost-effectiveness. In the context of LAD lesions, the application of HD-corrected FFR has demonstrated an increase in FFR values. This observation suggests a higher possibility of deferring PCI in such cases. Additionally, particularly in the context of non-invasive imaging-based methods such as CT-FFR, adjusting for the influence of HD in FFR is anticipated to improve the accuracy of FFR values. This adjustment can potentially contribute to reducing costs and risks associated with unnecessary invasive evaluations, including coronary angiography.

### Study limitations

This study has limitations. Firstly, the location of the Pd sensor was identified using CAG, while the HD was measured using CCTA tracing. Although measuring the HD on CAG is possible by turning the angle to the full lateral, this was not feasible in our retrospective study because a general CAG does not typically conduct such an angle projection. Additionally, using two-dimensional images in CCTA allows for identifying structural relationships and obtaining vertical heights based on the reference plane. Therefore, in this study, the Pd sensor location was inferred using CAG, and the HD was obtained from CCTA. Secondly, to obtain corrected FFR and Pd/Pa values, we used the corrected Pd formula derived from a previous study, as our formula could not be developed [9]. It is necessary to obtain an equation based on actual hydrostatic effect measurements in humans to ensure more accurate and reliable results in future studies. Thirdly, the clinical events during follow-up in patients with significant stenosis on corrected FFR, as compared to FFR alone, were not assessed in this study. Our future research endeavors will investigate the clinical outcomes that may differ based on corrected FFR.

## Conclusions

During FFR measurements, the hydrostatic pressure variations resulting from HD between the coronary ostium and the distal coronary pressure sensor could change the Pd and FFR values, affecting the evaluation of disease severity and treatment decisions. Further studies are needed to investigate the impact of HD on the FFR and its clinical significance.

## Impact on daily practice

FFR is calculated as the ratio of Pd to aortic pressure at peak hyperemia and Pd can be affected by hydrostatic pressure changes due to HD between the coronary ostium and the Pd sensor. In real-world data, HD can vary during intracoronary pressure measurements depending on coronary anatomy. The location of the Pd sensor, which was mostly higher than the ostium in the LAD, resulted in negative HD values. When corrected, the calculated FFR was significantly different from the original FFR value. In other words, HD can alter Pd and FFR values, which can affect disease severity assessment and treatment decisions.
